# Supplementary material for: Targeting the IL-6/STAT3 Signalling Cascade to Reverse Tamoxifen Resistance in Estrogen Receptor Positive Breast Cancer
Source: Cancers (Basel). 2021 Mar 25;13(7):1511. doi: 10.3390/cancers13071511 (PMC8036560; doi:10.3390/cancers13071511)
Supplement: Supplementary file 1 [file cancers-13-01511-s001.pdf]

cancers

MDPI

Supplementary Material: Targeting the IL-6/STAT3 Signalling Cascade to Reverse Tamoxifen Resistance in Estrogen Receptor Positive Breast Cancer

Ho Tsoi, Ellen P.S. Man, Ka Man Chau and Ui-Soon Khoo

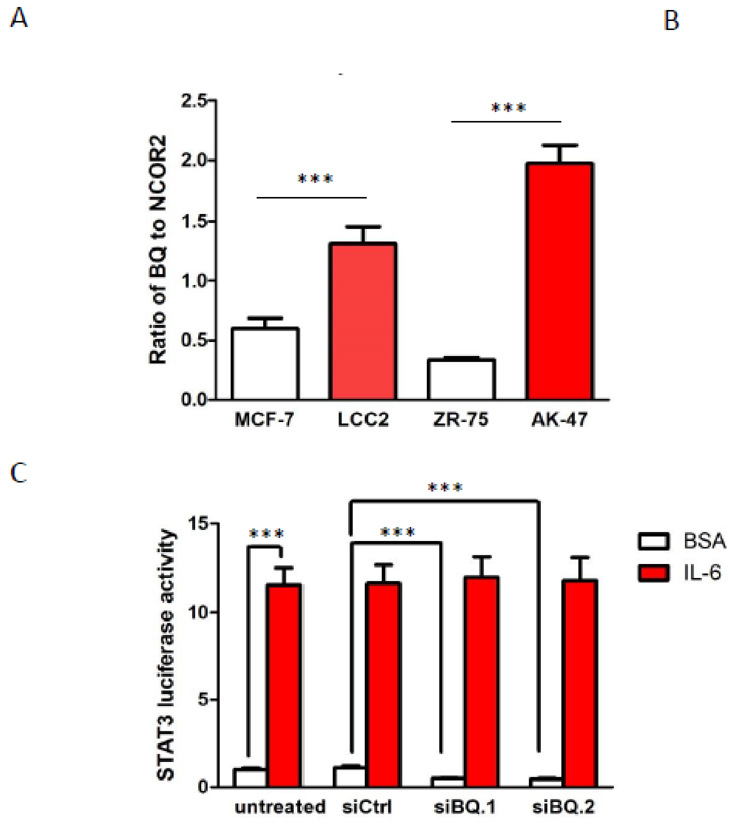

Figure S1. Supplementary information to Figure 1. (**A**) The ratio of BQ to NCOR2 in tamoxifen sensitive cell lines MCF-7 and ZR-75 was compared with that in tamoxifen resistant cell lines LCC2 and AK-47. LCC2 and ZK-47 were derived from MCF-7 and ZR-75 respectively. qPCR was employed to determine the expression of BQ and NCOR2. Actin was used as the internal control. Expression of BQ and NCOR2 in non-cancerous cell line MCF-10A was used as the reference. The relative expression of BQ and NCOR2 was determined and the ratio of BQ expression level to NCOR2 expression level was calculated. (**B**) Expression of NCOR2 was determined in LCC2 cells treated with siRNA against BQ. LCC2 cells were treated with 20 pmol of siCtrl, siBQ.1 or siBQ.2 for 72 hours. qPCR was employed. Actin was used as the internal control. Expression of NCOR2 in untreated LCC2 was used as the reference. (**C**) The addition of IL-6 could rescue the effect of BQ knockdown on STAT3 activity. 10 ng/mL of IL-6 or BSA was used. Luciferase reporter assay with STAT3 response element was employed. Results were shown as mean ± SD from at least three independent experiments. Students' *t* test was used for statistical analysis. *** represent $p < 0.001$.

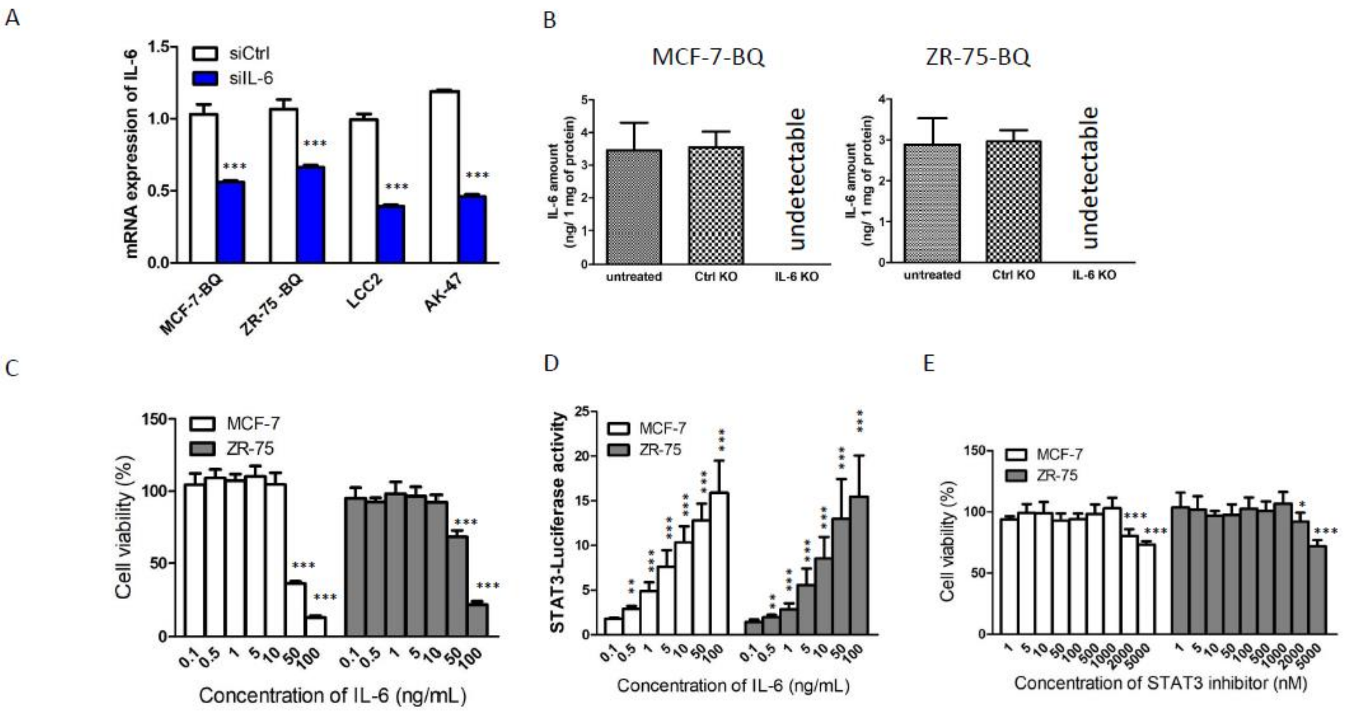

Figure S2. Supplementary information to Figure 2. (**A**) Knockdown efficiency of siRNA against IL-6 in breast cancer cell lines. 20 pmol of siRNA against IL-6 (siIL-6) and non-targeting siRNA were used. qPCR was performed 72 hours post transfection to determine the relative mRNA expression of IL-6. Untransfected cells were used as the reference. Actin was used as the internal control. (**B**) The effect of IL-6 knockout on the production of IL-6 in MCF-7-BQ and ZR-75-BQ. ELISA was performed on the cell lysates to determine the amount of IL-6. (**C**) The effect of different IL-6 concentration on cell viability of MCF-7 and ZR-75. MTT assay was performed after 72 hours of the treatment. The untreated cells were used as the reference. Statistical difference was compared with 0.1 ng/mL treatment group. (**D**) The effect of different IL-6 concentration on STAT3 activity of MCF-7 and ZR-75. Luciferase reporter assay with STAT3 response element was used. Untreated cells were used as the reference for fold change calculation. Renilla was used as the internal control. Statistical difference was compared with 0.1 ng/mL treatment group. (**E**) The effect of different STAT3 inhibitor S3I-201 concentration on cell viability of MCF-7 and ZR-75. MTT assay was performed after 72 hours of the treatment. The cells treated with DMSO were used as the reference. Statistical difference was compared with 1 nM treatment group. Results were shown as mean ± SD from at least three independent experiments. Students' t test was used for statistical analysis. *, **, *** represent $p < 0.05$, $p < 0.01$ and $p < 0.001$ respectively.

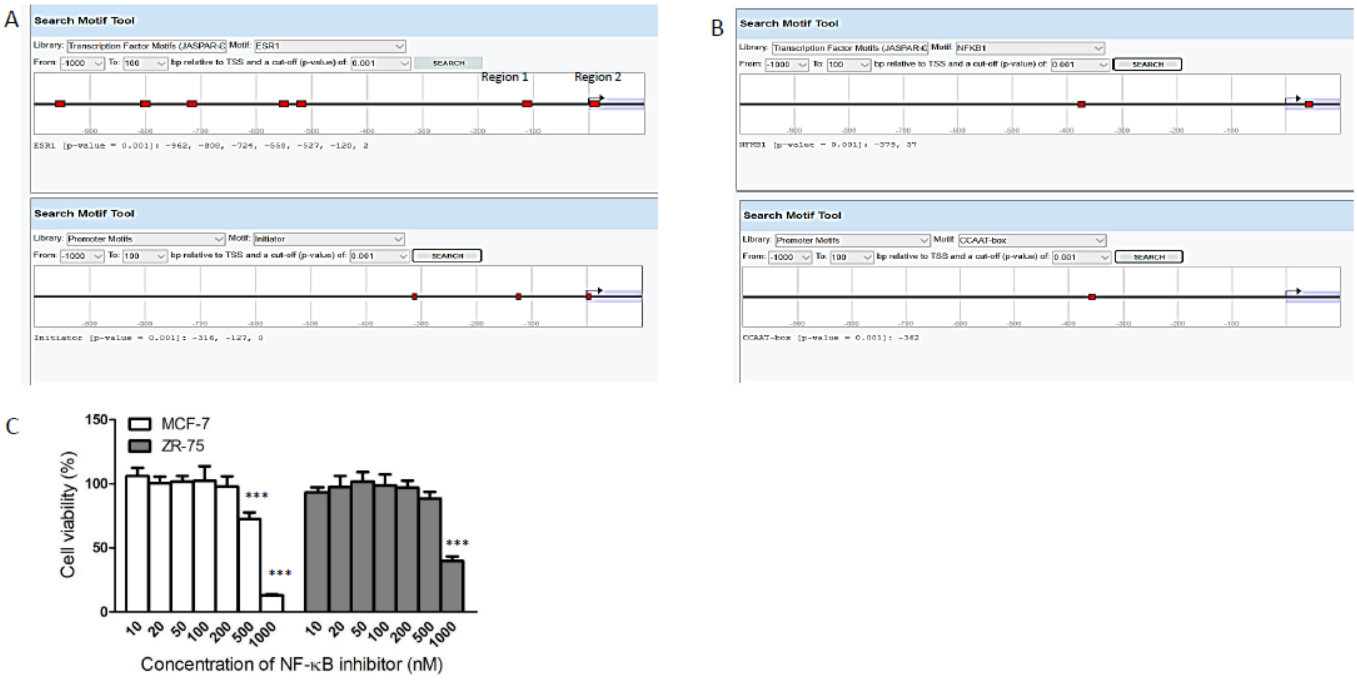

The Eukaryotic Promoter Database: expansion of EPDnew and new promoter analysis tools. Nucleic Acids Res. (2014); PMID: 25378343

Figure S3. Supplementary information to Figure 3. (**A**) Promoter analysis of IL-6. 7 potential ER binding sites (ERE) were identified. Only 2 ERE sites, region 1 and region 2, were close to the transcription initiator. (**B**) Promoter analysis of IL-6R. One potential p50 (NF-κB1) binding site (p50 RE) was identified to be close to the transcription initiator. (**C**) The effect of different NF-κB inhibitor pXSC concentrations on cell viability of MCF-7 and ZR-75. MTT assay was performed after 72 hours of the treatment. The cells treated with DMSO were used as the reference. Statistical difference was compared with 10 nM treatment group. Results were shown as mean ± SD from at least three independent experiments. Students' *t* test was used for statistical analysis. *** represents $p < 0.001$.

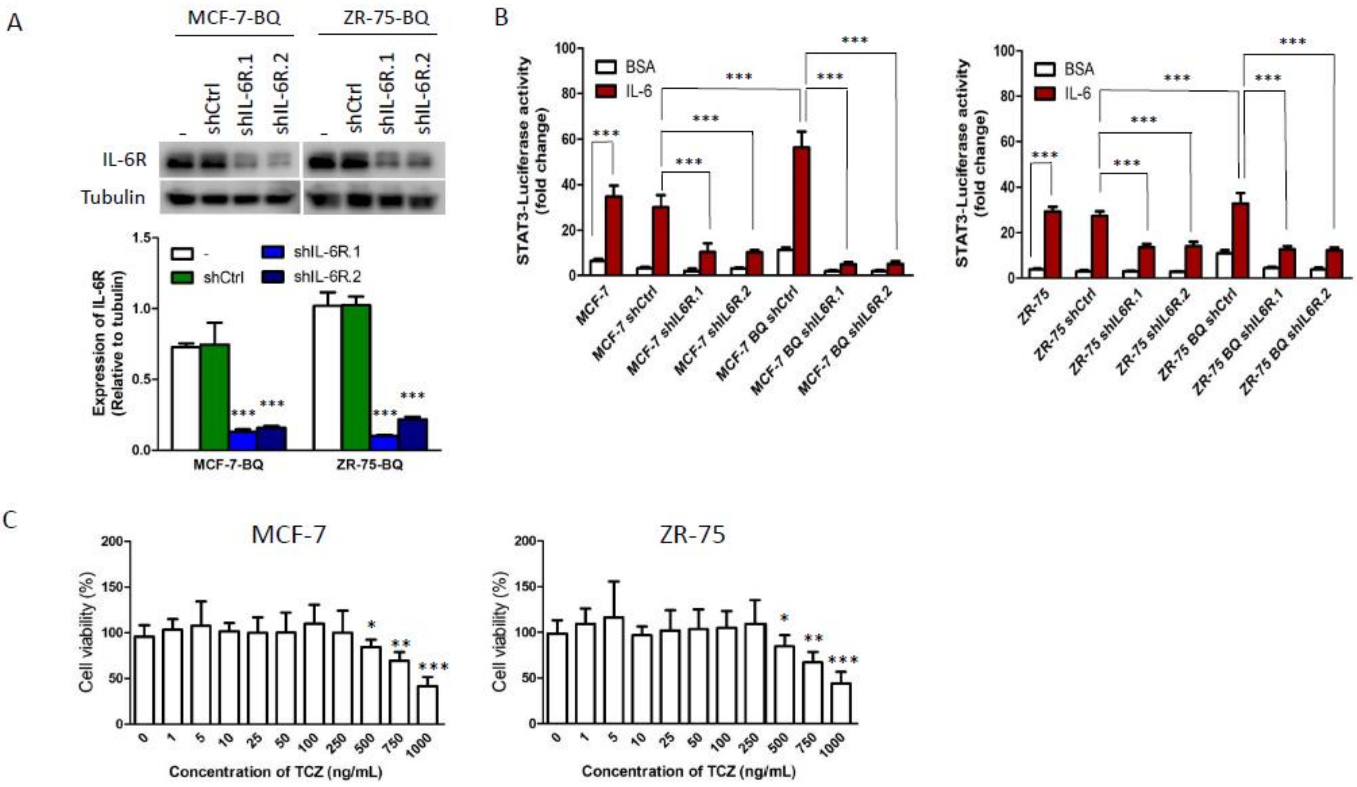

Figure S4. Supplementary information to Figure 4. (**A**) Knockdown effect of IL-6R. Stable cells lines MCF-7-BQ and ZR-75-BQ with IL-6R knockdown were established. shCtrl and shIL-6R represents non-targeting shRNA and IL-6R specific shRNA respectively. Two independent shIL-6R expressing plasmids were used. The expression of IL-6R was determined

by western blot. Tubulin was used as loading control. (**B**) The effect of IL-6 on STAT3 activity in different cell lines were determined. Luciferase reporter assay with STAT3 response element was used. Untreated cells were used as the reference for fold change calculation. Renilla was used as the internal control. (**C**) The effect of different Tocilizumab (TCZ) concentration on cell viability of MCF-7 and ZR-75. MTT assay was performed after 72 hours of the treatment. The cells treated with saline were used as the reference. Statistical difference was compared with 0 ng/mL treatment group. Results were shown as mean ± SD from at least three independent experiments. Students' t test was used for statistical analysis. *, **, *** represent $p < 0.05$, $p < 0.01$ and $p < 0.001$ respectively.

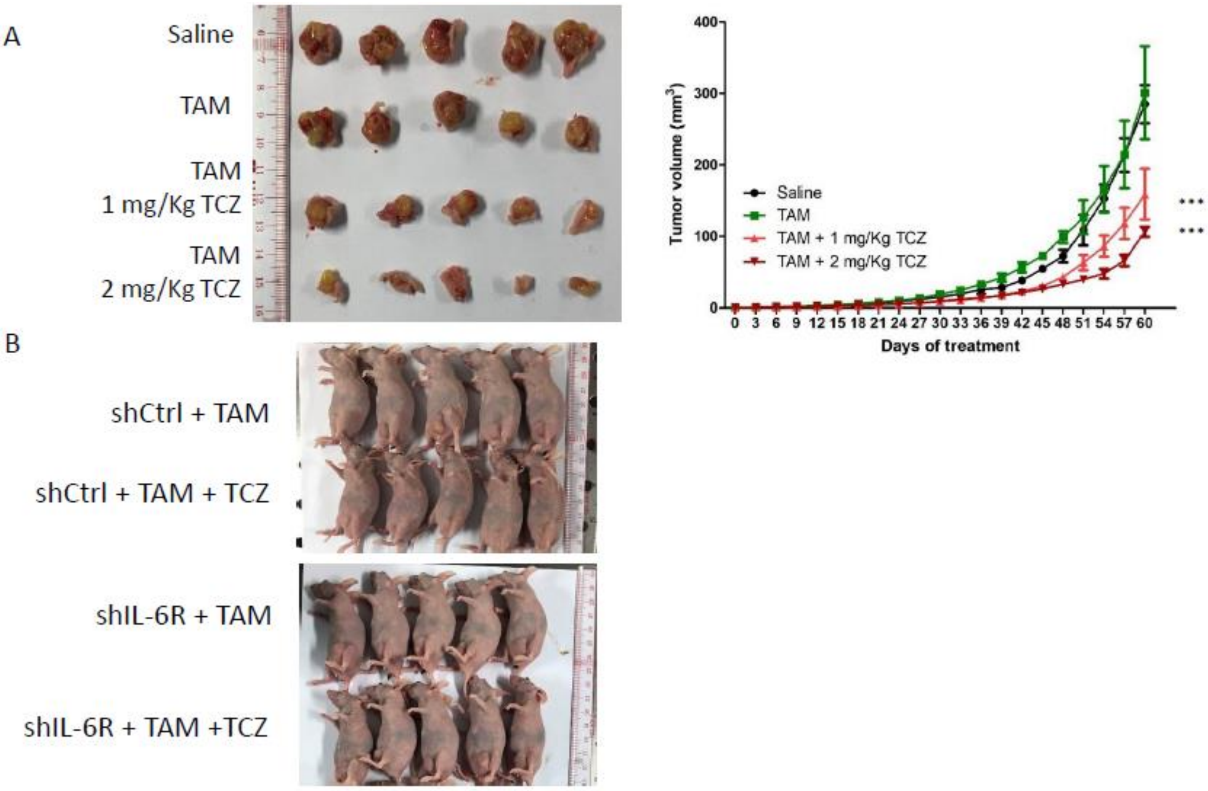

Figure S5. Supplementary information to Figure 5. (**A**) The effect of Tocilizumab (TCZ) on tamoxifen response. ZR-75-BQ cells were used to establish the xenograft model. The mice were treated with 0.5 mg of tamoxifen and 1 mg/Kg of TCZ or 2 mg/Kg of TCZ. Saline was used as solvent control. The mice were treated twice a week for 8 weeks. Tumor volume was recorded. Statistical difference was compared with saline treated group. Results were shown as mean ± SD from 5 independent mice. Students' *t* test was used for statistical analysis. (**B**) The images showing the tumor bearing nude mice.

Figure S6A

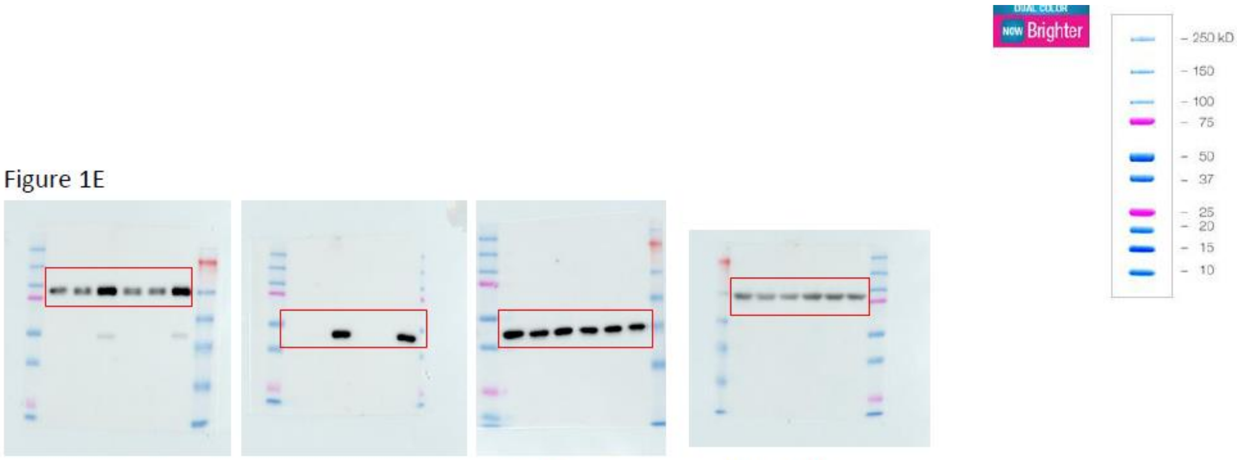

Anti- pSTAT3 (1:1,000: 9131, Cell Signaling Technology)

anti-HIS tag (1: 4,000; #2366; Cell Signaling Technology)

anti-actin (1:10,000; sc-47778; Santa Cruz Biotechnology)

Anti- STAT3 (1:1,000: 9139, Cell Signaling Technology)

Figure S6B

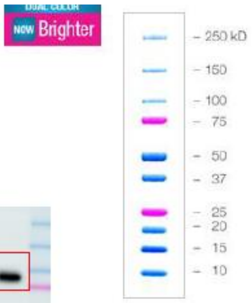

Figure 1G

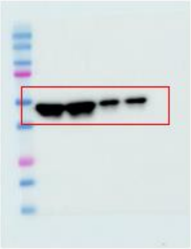

anti-BQ (1: 500 D-12; Versitech LTD)

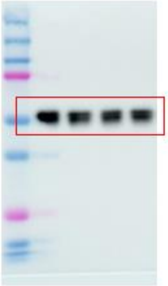

anti-tubulin (1:10,000; 2146; Cell Signaling Technology)

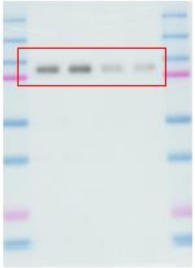

Anti- pSTAT3 (1:1,000: 9131, Cell Signaling Technology)

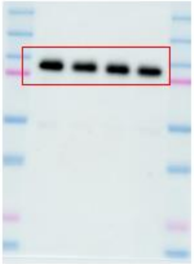

Anti- STAT3 (1:1,000: 9139, Cell Signaling Technology)

Figure S6C

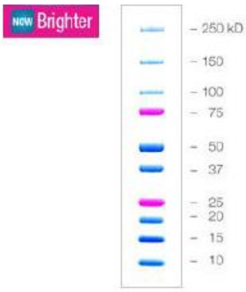

Figure 4B

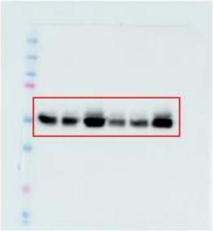

anti-IL-6R (1: 2,000; ab128008; Abcam)

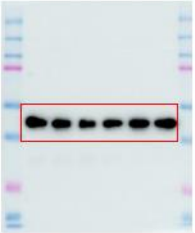

anti-actin (1:10,000; sc-47778; Santa Cruz Biotechnology)

Figure S6D

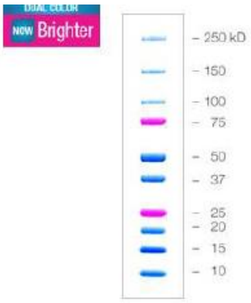

Figure 4D

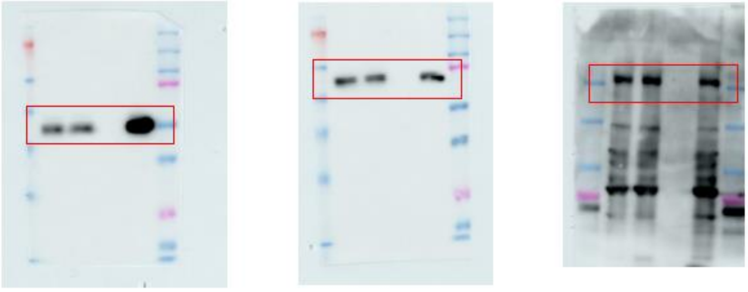

anti-p50 (1: 2,000; 13586; Cell Signaling Technology)

anti-p65 (1: 1,000; 8242; Cell Signaling Technology)

anti-NCOR2 (1: 1,000; ab24551l; Abcam)

Figure S6E

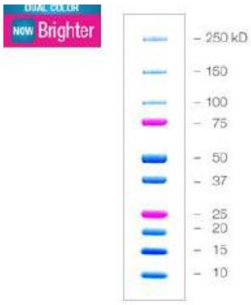

Figure 4E

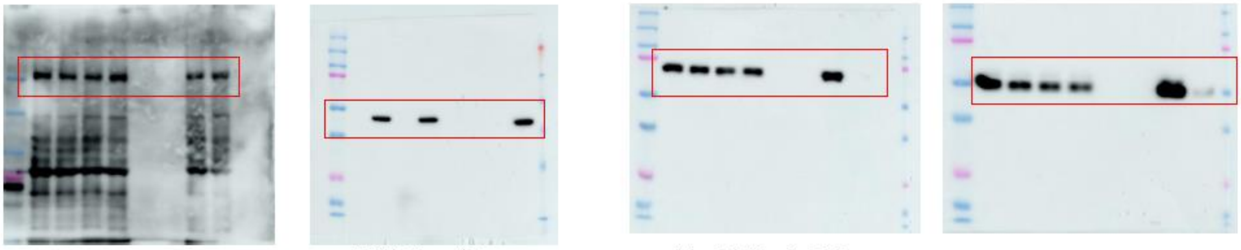

anti-NCOR2 (1: 1,000; ab24551l; Abcam)

anti-HIS tag (1: 4,000; #2366; Cell Signaling Technology)

anti-p65 (1: 1,000; 8242; Cell Signaling Technology)

anti-p50 (1: 2,000; 13586; Cell Signaling Technology)

Figure S6. Uncropped blots used in main figures. (**A**) Uncropped blot of Figure 1E. (**B**) Uncropped blot of Figure 1G. (**C**) Uncropped blot of Figure 4B. (**D**) Uncropped blot of Figure 4D. (**E**) Uncropped blot of Figure 4E.

Figure S7

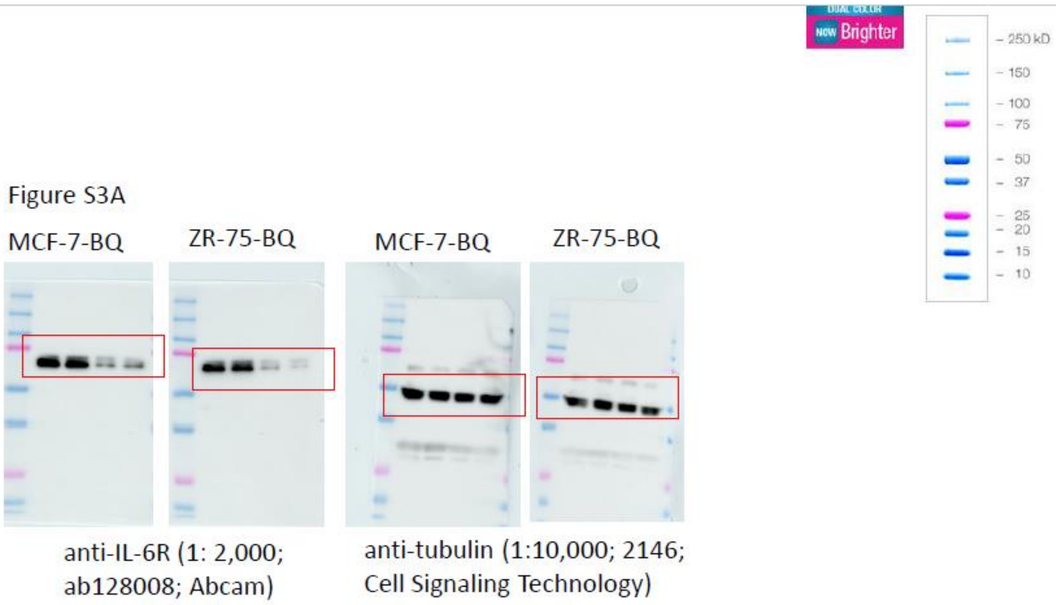

Figure S7. Uncropped blots used in supplementary figures. Uncropped blot of Figure S3A.

Table S1. The effect of BQ on the expression of candidate genes in the PCR array.

	MCF-7	MCF-7-BQ	MCF-7-BQ/MCF-7	Log2 (MCF-7-BQ/MCF-7)	ZR-75	ZR-75-BQ	ZR-75-BQ/ZR-75	Log2 (ZR-75-BQ/ZR-75)
	Relative to actin				Relative to actin			
AKT1	0.001	0.008	7.586	2.923	0.002	0.012	7.781	2.960
ANGPT1	0.000	0.001	1.107	0.147	0.000	0.000	1.811	0.857
ANGPT2	0.001	0.001	1.028	0.040	0.000	0.000	2.114	1.080
ANGPTL4	0.008	0.035	4.317	2.110	0.001	0.001	0.853	-0.230
APAF1	0.008	0.008	0.979	-0.030	0.003	0.003	1.023	0.033
BAD	0.035	0.035	0.993	-0.010	0.007	0.006	0.863	-0.213
BAX	0.009	0.009	0.993	-0.010	0.003	0.003	0.924	-0.113
BCL2	0.035	0.183	5.302	2.407	0.012	0.055	4.553	2.187
BCL2L1	0.126	0.118	0.933	-0.100	0.013	0.057	4.377	2.130
BRCA1	0.002	0.002	0.964	-0.053	0.007	0.006	0.875	-0.193
CASP8	0.000	0.000	0.485	-1.043	0.000	0.000	0.984	-0.023
CCNE1	0.000	0.001	3.458	1.790	0.002	0.007	4.307	2.107
CDC25A	0.004	0.004	1.099	0.137	0.007	0.007	0.991	-0.013
CDK2	0.001	0.033	32.297	5.013	0.002	0.004	2.479	1.310
CDK4	0.004	0.017	4.209	2.073	0.003	0.012	3.918	1.970
CDKN1A	0.001	0.000	0.362	-1.467	0.006	0.002	0.252	-1.987
CDKN2A	0.004	0.003	0.570	-0.810	0.002	0.001	0.468	-1.097
CFLAR	0.001	0.001	1.079	0.110	0.000	0.000	0.948	-0.077
CHEK2	0.002	0.001	0.215	-2.220	0.001	0.001	1.002	0.003
COL18A1	0.001	0.001	0.831	-0.267	0.001	0.001	0.998	-0.003
E3F1	0.003	0.004	1.245	0.317	0.002	0.001	0.779	-0.360

EPDR1	0.001	0.001	1.055	0.077	0.000	0.000	1.954	0.967
ERBB2	0.004	0.001	0.196	-2.350	0.002	0.003	1.862	0.897
ETS2	0.002	0.002	0.841	-0.250	0.001	0.000	0.219	-2.190
FGFR2	0.000	0.001	2.292	1.197	0.003	0.015	4.469	2.160
FOS	0.001	0.001	1.117	0.160	0.002	0.003	2.245	1.167
HIF1A	0.004	0.067	16.186	4.017	0.003	0.055	16.261	4.023
HK2	0.004	0.035	9.669	3.273	0.013	0.108	8.225	3.040
HTATIP2	0.004	0.003	0.705	-0.503	0.002	0.034	21.210	4.407
IFNA1	0.008	0.007	0.927	-0.110	0.004	0.015	4.141	2.050
IFNB1	0.001	0.001	0.910	-0.137	0.003	0.014	4.801	2.263
IGF1	0.002	0.002	1.102	0.140	0.007	0.028	3.991	1.997
ITGA1	0.002	0.002	0.998	-0.003	0.007	0.002	0.243	-2.040
ITGAV	0.001	0.001	1.030	0.043	0.002	0.002	1.136	0.183
ITGB1ITGB3	0.000	0.000	0.474	-1.077	0.007	0.002	0.243	-2.040
ITGB5	0.002	0.002	0.820	-0.287	0.007	0.002	0.259	-1.947
JUN	0.000	0.001	1.094	0.130	0.006	0.007	1.110	0.150
LDHA	0.002	0.016	8.074	3.013	0.003	0.028	9.426	3.237
LOX	0.002	0.004	2.099	1.070	0.002	0.012	6.837	2.773
MAP2K1	0.004	0.003	0.742	-0.430	0.013	0.006	0.474	-1.077
MCAM	0.008	0.009	1.033	0.047	0.006	0.002	0.270	-1.887
MDM2	0.001	0.008	7.621	2.930	0.004	0.012	3.182	1.670
MET	0.001	0.002	1.936	0.953	0.014	0.045	3.257	1.703
MMP1	0.001	0.002	2.000	1.000	0.012	0.042	3.403	1.767
MMP2	0.001	0.002	2.250	1.170	0.013	0.041	3.038	1.603
MMP9	0.004	0.004	0.979	-0.030	0.002	0.002	1.115	0.157
MTA1	0.010	0.011	1.079	0.110	0.001	0.002	1.149	0.200
MTA2	0.002	0.000	0.231	-2.117	0.002	0.002	1.072	0.100
MTSS1	0.009	0.008	0.920	-0.120	0.002	0.002	1.009	0.013
MYC	0.001	0.008	7.413	2.890	0.002	0.012	7.243	2.857
NFKB1	0.035	0.071	2.019	1.013	0.003	0.001	0.272	-1.880
NFKB1A	0.002	0.004	1.879	0.910	0.006	0.001	0.227	-2.137
NME1	0.000	0.000	0.998	-0.003	0.003	0.014	4.056	2.020
NME4	0.000	0.001	1.186	0.247	0.003	0.013	3.873	1.953
PDGFA	0.017	0.017	0.961	-0.057	0.028	0.009	0.322	-1.637
PDGFB	0.008	0.008	1.002	0.003	0.000	0.000	1.030	0.043
PIK3R1	0.002	0.009	4.563	2.190	0.003	0.015	4.868	2.283
PLAU	0.001	0.001	0.929	-0.107	0.004	0.007	1.941	0.957
PLAUR	0.001	0.001	0.984	-0.023	0.012	0.012	0.973	-0.040
PNN	0.000	0.000	0.887	-0.173	0.002	0.002	1.136	0.183
RAF1	0.010	0.007	0.725	-0.463	0.007	0.002	0.243	-2.040
RB1	0.005	0.009	1.794	0.843	0.059	0.029	0.487	-1.037
RBPJ	0.001	0.001	1.125	0.170	0.007	0.002	0.259	-1.947
S100A4	0.116	0.130	1.120	0.163	0.099	0.007	0.066	-3.930
S100A4	0.002	0.001	0.566	-0.820	0.056	0.055	0.986	-0.020
SERPINB5	0.002	0.001	0.555	-0.850	0.014	0.014	0.986	-0.020
SERPINE1	0.002	0.001	0.531	-0.913	0.006	0.029	4.834	2.273
SNCG	0.037	0.124	3.317	1.730	0.006	0.002	0.270	-1.887
SYK	0.000	0.000	1.823	0.867	0.002	0.002	1.045	0.063

TEK	0.008	0.008	1.072	0.100	0.000	0.000	0.824	-0.280
TERT	0.037	0.028	0.770	-0.377	0.026	0.011	0.448	-1.160
TGFB1	0.008	0.008	0.986	-0.020	0.001	0.000	0.651	-0.620
TGFBR1	0.017	0.006	0.325	-1.620	0.002	0.002	0.961	-0.057
THBS1	0.018	0.005	0.301	-1.733	0.001	0.002	1.067	0.093
TIMP1	0.001	0.001	1.002	0.003	0.000	0.000	0.482	-1.053
TIMP3	0.001	0.000	0.859	-0.220	0.001	0.002	1.072	0.100
TNF	0.018	0.006	0.324	-1.627	0.002	0.002	1.167	0.223
TNFRSF10B	0.001	0.000	0.116	-3.113	0.002	0.002	0.995	-0.007
TNFRSF1A	0.004	0.000	0.059	-4.073	0.003	0.003	0.825	-0.277
TNFRSF25	0.008	0.004	0.494	-1.017	0.006	0.006	0.986	-0.020
TP53	0.008	0.007	0.861	-0.217	0.014	0.006	0.453	-1.143
TWIST1	0.004	0.004	1.050	0.070	0.000	0.000	0.869	-0.203
USF2	0.002	0.008	4.028	2.010	0.000	0.001	2.707	1.437
VEGF	0.000	0.004	8.225	3.040	0.007	0.027	4.151	2.053

Table S2 Affected genes by BQ overexpression in each of the pathways.

Pathways	Affected members in the pathway
IL6-mediated signaling events	MYC, TIMP1, JUN, PIK3R1, FOS, BCL2L1, AKT1
FGF signaling pathway	FOS, MET, JUN, AKT1, PLAU, PLAUR, FGFR2, PIK3R1, MMP9
Central carbon metabolism in cancer	MYC, LDHA, MAP2K1, RAF1, MET, AKT1, ERBB2, HIF1A, HK2, TP53, FGFR2, PIK3R1
Angiogenesis	TEK, PDGFB, TIMP3, AKT1, HIF1A, FGFR2, MMP9
HIF-1 signaling pathway	CDKN1A, LDHA, TIMP1, HK2, TEK AKT1, ERBB2, HIF1A, BCL2, VEGF, IGF1, MAP2K1, PIK3R1, ANGPT1, ANGPT2, SERPINE1
LPA receptor mediated events	AKT1, IGF1, MMP2, PIK3R1, MMP9
PI3K-Akt signaling pathway	IFNB1, MYC, CDKN1A, RAF1, MET, CCNE1, THBS1, IGF1, ANGPT1, ANGPT2, BRCA1, PDGFA, PDGFB, PIK3R1, NFKB1, BAD, SYK, AKT1, BCL2, BCL2L1, FGFR2, ITGA1, MDM2, TEK, MAP2K1, ITGAV, ITGB5, IFNA1, TP53, CDK2, CDK4
